# Workplace-based assessment: raters’ performance theories and constructs

**DOI:** 10.1007/s10459-012-9376-x

**Published:** 2012-05-17

**Authors:** M. J. B. Govaerts, M. W. J. Van de Wiel, L. W. T. Schuwirth, C. P. M. Van der Vleuten, A. M. M. Muijtjens

**Affiliations:** 1 Department of Educational Research and Development, FHML, Maastricht University, P.O. Box 616, 6200 MD Maastricht, The Netherlands; 2 Department of Work and Social Psychology, FPN, Maastricht University, Maastricht, The Netherlands; 3 School of Medicine, Flinders University, GPO Box 2100, Adelaide, SA 5001 Australia

**Keywords:** Clinical education, Cognition-based assessment models, Competence assessment, Performance assessment, Professional judgment, Rater judgments, Rating process, Workplace-based assessment

## Abstract

Weaknesses in the nature of rater judgments are generally considered to compromise the utility of workplace-based assessment (WBA). In order to gain insight into the underpinnings of rater behaviours, we investigated how raters form impressions of and make judgments on trainee performance. Using theoretical frameworks of social cognition and person perception, we explored raters’ implicit performance theories, use of task-specific performance schemas and the formation of person schemas during WBA. We used think-aloud procedures and verbal protocol analysis to investigate schema-based processing by experienced (N = 18) and inexperienced (N = 16) raters (supervisor-raters in general practice residency training). Qualitative data analysis was used to explore schema content and usage. We quantitatively assessed rater idiosyncrasy in the use of performance schemas and we investigated effects of rater expertise on the use of (task-specific) performance schemas. Raters used different schemas in judging trainee performance. We developed a normative performance theory comprising seventeen inter-related performance dimensions. Levels of rater idiosyncrasy were substantial and unrelated to rater expertise. Experienced raters made significantly more use of task-specific performance schemas compared to inexperienced raters, suggesting more differentiated performance schemas in experienced raters. Most raters started to develop person schemas the moment they began to observe trainee performance. The findings further our understanding of processes underpinning judgment and decision making in WBA. Raters make and justify judgments based on personal theories and performance constructs. Raters’ information processing seems to be affected by differences in rater expertise. The results of this study can help to improve rater training, the design of assessment instruments and decision making in WBA.

## Introduction

Observation and assessment of trainee performance in ‘real-life’ professional settings has been a cornerstone of health professions education for centuries. It is the potentially best way of collecting data and providing feedback on what trainees actually *do* in day-to-day practice. Indeed, current assessment practices are characterized by growing emphasis on workplace-based assessment (WBA), stimulated by the widespread implementation of competency-based curricula, increasing demands for physician accountability and concerns about health care quality as well as calls for improved supervision and assessment of medical trainees (Davies 2005; Norcini 2005; Kogan et al. 2009; Holmboe et al. 2010).

Although there is general agreement that WBA is useful for formative assessment, its usefulness for summative assessment is not undisputed (Norcini and Burch 2007; McGaghie et al. 2009). Major concerns about the utility of WBA relate to its inherent subjectivity and the resulting weaknesses in the quality of measurement. In general, the idiosyncratic nature of (untrained) rater judgments results in large differences between performance ratings, low inter- and intra-rater reliabilities and questionable validity of WBA (Albanese 2000; Williams et al. 2003). More to the point, research into performance appraisals in various domains suggests that idiosyncratic rater effects account for substantial variance in performance ratings, ranging from 29 % to over 50 % (Viswesvaran et al. 1996; Scullen et al. 2000; Hoffman et al. 2010). Consequently, attempts to improve WBA tend to focus on minimizing the ‘subjectivity factor’ through standardization of assessment procedures and rater training. However, such measures have met with mixed success at best (Williams et al. 2003; Lurie et al. 2009; Holmboe et al. 2010; Green and Holmboe 2010).

Research findings suggest many reasons why rater behaviour may be quite impervious to change despite training and/or the use of worked out (detailed) assessment tools. Research in industrial and organizational psychology, for instance, indicates that raters often have implicit performance theories, which may diverge from those specified by the organization (Borman 1987; Ostroff and Ilgen 1992; Uggerslev and Sulsky 2008). Research furthermore indicates that rating outcomes are determined by a complex and interrelated set of factors in the social setting of the assessment process, such as local norms and values, time pressure, assessment goals and affective factors (Murphy and Cleveland 1995; Levy and Williams 2004). Recent research by Ginsburg et al. (2010) suggests that also in the medical domain assessment tools and theoretical models of professional competence may not adequately reflect supervisors’ theories of work performance, resulting in ‘blurring’ of competency domains and seemingly invalid or inaccurate (“less authentic”) performance ratings. In other words, there may very well be discrepancies between how we feel that raters *should* think or act (theory espoused) and what they actually think and *do* in practice (theory in use). Similarly, Holmboe et al. (2010) state that in fact “… we know very little about effective faculty observation skills and behaviors”.

In order to effectively improve WBA, we clearly need a better understanding of the underpinnings of rater behaviours in the context of WBA and, as suggested by Ginsburg et al., it may make sense to start by investigating what raters actually observe, experience and can comment on. The purpose of this study was to investigate how raters in WBA form impressions of and make judgments on trainee performance. More specifically, we explored whether theoretical frameworks of social perception can be used to further our understanding of processes underlying judgment and decision making in performance assessments in the clinical setting, so as to improve the utility of assessment outcomes.

## Conceptual framework

### Raters as social perceivers

It is inherent in WBA that all information must ultimately pass the cognitive filter represented by the rater (Landy and Farr 1980; Smith and Collins 2009). This implies that understanding the evaluation of performance in real life is basically about understanding how raters form impressions and make inferences (e.g. judgments and decisions) about other people in interpersonal and social environments. Indeed, it is increasingly recognized that raters are to be seen as ‘social perceivers’ providing ‘motivated social judgments’ when evaluating performance (Murphy and Cleveland 1995; Klimoski and Donahue 2001; Levy and Williams 2004). A central assumption in this approach is that raters are active information processors who, within a dynamic and complex social setting, are faced with the cognitive tasks of gathering, interpreting, integrating and retrieving information for judgment and decision making (DeNisi 1996; Klimoski and Donahue 2001; McGaghie et al. 2009). Raters’ information processing is influenced by their understanding of (in)effective performance, personal goals, interactions with the ratee and others, as well as by other factors in the social context of the assessment process (Uggerslev and Sulsky 2008; Murphy et al. 2004; Govaerts et al. 2007). This view of how raters perceive and judge performance can be cast in theoretical frameworks of social perception as an element of social cognition. In fact, performance assessment might be seen as a ‘specific application of social perception for specific purposes, and much of raters’ behaviours can be considered to be rooted in social perception phenomena’ (Klimoski and Donahue 2001; Barnes-Farrell 2001).

### Performance assessment and social perception

Findings from social perception research consistently indicate that, when forming impressions and making judgments of others, social perceivers tend to use pre-existing knowledge structures, or ‘schemas’. Schemas can be thought of as adaptive mechanisms that enable people to efficiently process information, especially in situations where information is incomplete, ambiguous or where there are situational constraints (e.g. time pressure, conflicting tasks). In social perception most people use *role,* *event* and *person* schemas (Pennington 2000, pp. 69–75). A role schema can be defined as the sets of behaviours expected of a person in *a certain social position* (e.g. a policeman, teacher, family physician). Event schemas describe what we normally expect from other people’s behaviours *in specific social situations,* related to the predicted sequence of events in such a situation (e.g. a job interview or performance appraisal interview). Person schemas reflect the inferences we make about someone on the basis of (limited) available information, as we get to know them through verbal and non-verbal cues in their behaviour. Person schemas may include expected patterns of behaviour, personality traits and other inferences, such as conclusions about someone’s knowledge base or social category (for instance, ‘excellent performer’ or ‘poor performer’). When we observe others, these schemas together guide the focus of our attention, what we remember and how we use information in forming impressions and making judgments. The three types of schema should not be regarded as entirely distinct or separate: schemas are used interactively when we are trying to understand how people behave (Pennington 2000).

Key features of the framework we have described can easily be translated to the context of work-based performance assessment.

Firstly, the literature (e.g. Borman 1987; Ostroff and Ilgen 1992; Uggerslev and Sulsky 2008; Ginsburg et al. 2010) suggests that raters in work settings develop personal constructs or ‘theories’ of effective job performance in general. These ‘performance theories’ are very similar to role schemas in that they include sets or clusters of effective behaviours in relation to any number of performance dimensions considered relevant to the job. Since performance theories develop through (professional) experience, socialization and training, the content of performance theories is likely to vary between raters, resulting in varying levels of rater idiosyncrasy (Uggerslev and Sulsky 2008).

Secondly, research findings indicate that the particular set of behaviours related to effective performance may differ from one task to another, depending on the setting and specific features of the task (e.g. Veldhuijzen et al. 2007). Veldhuijzen et al. (2007), for instance, showed that physicians use different communication strategies depending on situational demands. It is therefore to be expected that, as a result of prolonged job experience, raters develop highly differentiated performance schemas, each representing different sets of effective behaviours for various and differentiated job-related tasks and task settings. When raters are observing others during task performance, task- or situation-specific cues may trigger the use of task- or event-specific schemas to judge performance, especially in more experienced raters.

Finally, when observing performance for assessment purposes, raters will inevitably develop ‘person schemas’ to organize their knowledge about individual ratees. Raters interpret observations, integrate information, and make inferences, for instance about a ratee’s knowledge base, level of competence or behavioural disposition.

When making judgments and decisions about performance by others, raters are likely to use all three schema types interactively: raters’ personal performance theory (‘role schema’), normative expectations of task-specific behaviours (task-specific schema) and inferences about the ratee (person schema) may all influence assessment outcomes (Cardy et al. 1987; Borman 1987).

### The present study

The objectives of the present study were to explore whether the social perception framework can be used to describe and explain cognitive processes underlying rater judgments in WBA. More specifically, we investigated the use and content of schemas by physician-raters when assessing trainee performance in patient encounters. Given research findings indicating the impact of rater effects on outcomes in work-based assessment (Hoffman et al. 2010; Govaerts et al. 2011), we additionally investigated differences between raters with respect to the performance dimensions used in judgment and decision making (rater idiosyncrasy) and how differences in rating experience affected schema-based processing. We used a mixed methods approach to address our research objectives. Qualitative verbal protocol analysis was used to explore:

- Raters’ performance theories (implicit role schemas);
- Raters’ use of task-specific performance schemas; and
- Raters’ formation of person schemas during observation and assessment of performance.

Quantitative analyses were used to investigate differences between raters.

## Method

### Participants

The participants in our study were GP-supervisors who were actively involved in supervising and assessing postgraduate trainees in general practice. The Dutch postgraduate programmes in general practice have a long tradition of systematic direct observation and assessment of trainee performance throughout the training programme. The participants in the study were all experienced GPs who supervised trainees on a day-to-day basis and were trained in observation and evaluation of trainee performance.

Registered GP-supervisors with different levels of experience were invited by letter to voluntarily participate in our study. A total of 34 GP-supervisors participated. In line with findings from expertise research (e.g. Arts et al. 2006), GP-supervisors with at least 7 years of experience as a supervisor-rater were defined as ‘experts’. The ‘expert group’ consisted of eighteen GPs (experience in general practice: *M* = 26.3 years; *SD* = 5.0 years; supervision experience: *M* = 13.4 years; *SD* = 5.9 years); the ‘non-expert group’ consisted of sixteen GP-supervisors (experience in general practice: *M* = 12.9 years; *SD* = 5.0 years; supervision experience: *M* = 2.6 years; *SD* = 1.2 years). Participants received financial compensation for their participation.

### Research procedure and data collection

Participants watched two video cases (VCs), each showing a sixth-year medical student in a ‘real-life’ encounter with a patient. The participants had not met the students before the study. The VCs were selected purposively to present common patient problems and different student performance. Both VCs presented ‘straightforward’ cases that are common in general practice: atopic eczema and angina pectoris. These cases were selected to ensure that all participants (both experienced and inexperienced raters) were familiar with the task-specific performance requirements. VC1 (atopic eczema) lasted about 6 min and presented a student showing prototypical and clearly substandard performance with respect to communication and interpersonal skills. VC2 (angina pectoris) lasted about 18 min and presented a student showing complex, i.e. more differentiated, performance with respect to both communication and patient management. Permission to record the patient encounter and to use the recording for research purposes was obtained from both students and patients.

Participants’ cognitive performance was captured through verbal protocol analysis (Chi 1997). Before the first video was started, the participants were informed about the research procedures and given a set of verbal instructions. They were specifically asked to ‘think aloud’ and to verbalize all their thoughts as they emerged, as if they were alone in the room. If a participant was silent for more than a few seconds, the research assistant asked him or her to continue. Permission to audiotape the sessions was obtained.

For both VCs the procedure was as follows:

1. The video is started. The participant signals when he or she feels able to judge the student’s performance; the video is then stopped (T1). The participant verbalizes his/her first judgment of the student’s performance (verbal protocol (VP) 1).
2. The participant gives an overall rating of performance on a one-dimensional rating scale (Fig. 1), thinking aloud while filling in the rating form (VP2).

**Fig. 1:**
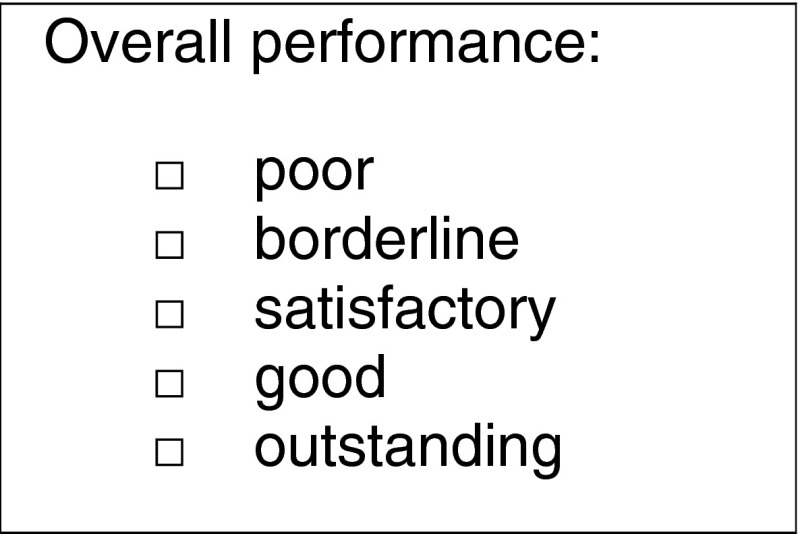
Rating form; 1-dimensional *overall* performance rating (VC procedure step 2)
3. The video is resumed at the point where it was stopped at T1. When the video ends (T2), the participant verbalizes his/her judgment (VP3) while giving a final overall rating.

We used a balanced design to control for order effects; the participants in the groups of expert and non-expert raters were alternately assigned to one of two viewing conditions, which differed in the order of presentation of the VCs.

### Data analysis

All audiotapes were transcribed verbatim. Verbal data were analyzed qualitatively (to explore the schemas the raters used in assessing performance) and quantitatively (to assess differences between raters and rater groups in the use of schemas).

### Qualitative analysis

We first performed bottom-up open coding of all protocols (VP1, VP2, and VP3 pooled) to explore the raters’ performance theories and task-specific performance schemas (Elo and Kyngäs 2008; Thomas 2006). Two researchers with different professional backgrounds (MG, MD-medical educator and MvdW, cognitive psychologist) coded transcripts independently, using an open thematic type of analysis to determine which performance dimensions raters used in judging trainee performance. The researchers met repeatedly to compare and discuss emergent coding structures until the coding framework was stable. The final coding framework, which was considered to represent raters’ aggregate performance theory (i.e. the set of dimensions used by the raters to evaluate performance) and the coding structures reflecting the use of task-specific schemas, was discussed with an experienced GP in order to assess confirmability (Barbour 2001; Kitto et al. 2008). This discussion did not result in any further changes of the coding structure.

We used top-down, a priori coding to explore the use of person schemas. The coding categories for ‘person schemas’ were based on the theoretical framework proposed by Klimoski and Donahue (2001), describing five common types of inference processes in judgment tasks: inferences regarding knowledge, traits, dispositions (probable patterns of behaviour), intentions (immediate goals) and social category membership. We added a separate category to this framework, indicating the use of ‘training phase’ as a frame of reference in making judgments.

Table 1 presents the final coding framework, which was applied to all verbal protocols using software for qualitative data analysis (Atlas-ti 6.1).

**Table 1:** Verbal protocol coding structures

**Performance theory:** performance dimensions and sub dimensions
1. Think and act like a general practitioner
2. Doctor-patient relationship
2.1. Atmosphere
2.2. Balanced patient-centeredness
2.2.1. Develop and establish rapport
2.2.2. Demonstrate appropriate confidence
2.2.3. Demonstrate empathy/empathic behaviour, appropriate for problem
2.2.4. Open approach
2.2.5. Facilitating shared mind 1 = identifying reasons for consultation; exploring patient’s perspective
2.2.6. Facilitating shared mind 2 = explain rationale for questions, examinations; explain process; share own thinking
2.2.7. Facilitating shared mind 3 = collaborative decision making
3. Handling (bio)medical aspects (disease)
3.1. History
3.2. Physical examination
3.3. Diagnosis/differential diagnosis
3.4. Patient management plan
4. Structuring of the consultation and time management
**Task- (event-)specific schema**
1. Identification of case-specific cues
1.1. Specific aspects of the patient’s problem/clinical presentation (e.g. this type of eczema poses very serious social problems to the patient)
1.2. Specific aspects of the patient’s behaviours (verbal as well as non-verbal; e.g. this patient is very talkative)
1.3. Setting/context of the medical consultation (GP’s office versus outpatient clinic)
2. Trainee behaviours (effective or ineffective) within performance domain X, explicitly related to case-specific cues
3. Effects of trainee behaviour on patient behaviour/doctor-patient relationship (positive or negative)
**Person schema**
1. Inferences regarding
1.1. Knowledge base
1.2. Personality traits (e.g. he is a very nice guy)
1.3. Disposition (e.g. this trainee has a clinical method of working; finds it difficult to just lean back and listen to what patients are saying)
1.4. Intention (e.g. he seems to be focused on the biomedical aspect of the patient’s problem)
1.5. Category (e.g. he is an authoritarian doctor; he will become an excellent doctor)
2. Phase of training (frame of reference for making judgments)

### Quantitative analysis

In order to explore differences between raters in the use of performance theories and task-specific performance schemas, the verbal protocols were reanalyzed using the coding framework as presented in Table 1. For this analysis, VP1 and VP2 were merged to create a single verbal protocol containing all verbal utterances at T1. The transcripts of the verbal protocols were segmented into phrases by one of the researchers (MG). Each segment represented a single coherent thought or statement about the trainee or trainee performance (e.g. description of a particular behaviour within a performance dimension or a judgment remark about overall effectiveness on a particular performance dimension). Additionally, statements about trainee performance were coded along the dimension positive versus negative (i.e. effective versus ineffective behaviour). Repetitions were coded as such. Six randomly selected protocols were coded by two independent coders (MG, MvdW). Because inter-coder agreement was very high 90–100 %), the other protocols were coded by only one researcher (MG). Researchers met repeatedly, however, to discuss any uncertainties in coding. After coding, data were exported from Atlas.ti to SPSS 17.0. In order to explore rater idiosyncrasy with respect to the use of performance theory, we calculated, for each performance dimension, the percentage of raters using that performance dimension. Percentages were calculated for each VC at T1 and T2, respectively. Levels of rater idiosyncrasy in relation to any performance dimension can be inferred from the percentage of raters using that dimension, with 0 and 100 % indicating maximum inter-rater agreement, i.e. complete absence of idiosyncrasy, and 50 % indicating maximum disagreement, i.e. maximum level of idiosyncrasy. So, the closer the percentage moves to 50 %, the higher the level of idiosyncrasy. Additionally, the number of statements representing dimension-related performance (effective versus ineffective behaviours) was calculated for each of the performance dimensions.

Between-group differences in the use of task-specific schemas were estimated by transforming the number of statements per coding category per rater to percentages in order to correct for between-subject variance in verbosity and elaboration of answers. Because of the small sample sizes and non-normally distributed data, non-parametric tests (Mann–Whitney *U*) were used to estimate differences between the two groups. We calculated effect sizes using the formula *ES* = *Z*/√*N* as is suggested for non-parametric comparison of two independent samples, where *Z* is the *z*-score of the Mann–Whitney statistic and *N* is the total sample size (Field 2009, p. 550). Effect sizes of .1, .3 and .5 indicate a small, medium and large effect, respectively.

### Ethical approval

Dutch law and the Maastricht University IRB have considered this type of research exempt from ethical review. Pending the installation of a national Medical Education Research Review Board we have taken precautions to protect the interests of all participants (students, patients and GP-supervisors). Participation was voluntary and full confidentiality was guaranteed. Informed consent to record patient encounters and to use recordings for research purposes was obtained from the students and patients in the DVDs. Before we started data collection, all participants were informed about research procedures in writing, and permission to audiotape sessions was obtained. Data were analysed anonymously.

## Results

We first present the results of the qualitative data analysis, followed by the results of the quantitative analyses.

### Performance theory

Analysis of the verbal protocols resulted in identification of seventeen performance dimensions, used by the raters in assessing trainee behaviour during patient encounters. The raters distinguished four main dimensions (‘Think/act like a GP’, ‘doctor-patient relationship’, ‘handling of (bio)medical aspects’ and ‘structuring/time management’) and various sub-dimensions. Within the dimension ‘doctor-patient relationship’, two large sub-dimensions were identified. One sub-dimension included sets of behaviours relating to “creating a good atmosphere” for effective and efficient patient-doctor communication. This sub-dimension was considered by the raters at the beginning of the consultation in particular. The second sub-dimension (“balanced patient centeredness”) contains sets of behaviours facilitating patient involvement throughout the consultation while at the same time ensuring that the physician, as a professional medical expert, remains in charge of the consultation.

**Fig. 2:**
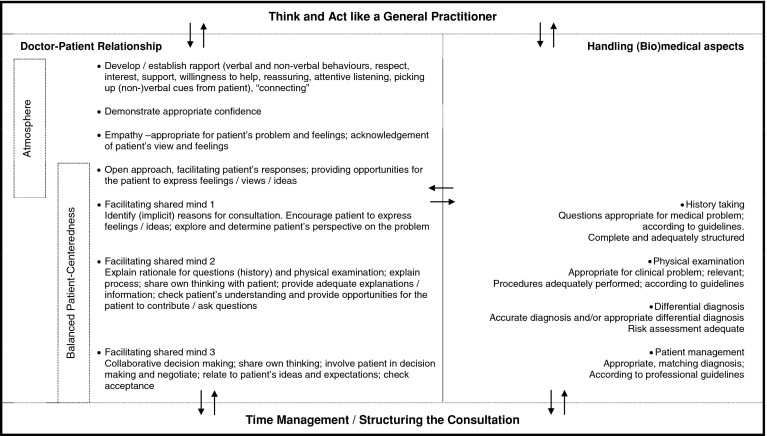
Aggregate performance theory, presenting the performance dimensions used by supervisor-raters in general practice when assessing trainee performance in GP patient encounters

The performance dimensions, their interrelationships and examples of performance- related behaviours are presented in Fig. 2.

Although participants clearly distinguished different dimensions, the data analysis also showed that they used dimensions interactively when judging performance effectiveness. When evaluating the doctor-patient relationship, for example, raters also considered whether the trainee organized and planned the consultation adequately:

*“In the beginning, he did very well to let the patient tell his story, but it took too long; he should have guided the patient in the right direction a bit sooner–although it is not bad at all to just sit and listen to what the patient has to say.” (PP 25)*

Similarly, when judging performance during physical examination, participants considered not only technical skills and smoothness in performing the examination, but also whether and how the trainee communicated with the patient before and during the examination and possible effects of the communication on the doctor-patient relationship:

*“Physical examination is also not so good.. takes blood pressure, palpates abdomen, auscultation of abdomen,.. okay,..but he examines the patient in dead silence. He doesn’t tell the patient what he is doing or what his findings are. This is not the way to gain the patient’s trust.” (PP 24)*

“*Technically, the physical examination seems adequate, but there was complete silence, no contact with the patient at all. … This can be another way to build trust with the patient, and it is not used at all.” (PP3)*

Also in judgments of history taking or patient management, behaviours within the dimension “doctor-patient relationship” were considered as important as ‘content-related’ behaviours within the dimension “(bio)medical aspects”.

*“His knowledge base seems adequate. From a cardiologic perspective the prescriptions are correct, but there is no connection at all with the patient’s view or feelings. … I therefore doubt whether he is able to think as a general practitioner.” (PP12)*

### Task-specific schema

Analysis of the verbal protocols resulted in three major categories reflecting the use of task-specific performance schemas (Table 1): identification of case-specific cues, identification of particular behaviours as (in)effective, explicitly in relation to case-specific cues, and effects of trainee behaviour on the particular patient. These categories represent comments that focus not only on discrete aspects of raters’ performance theory, but also explicitly and specifically link (in)effective behaviours and performance to case-specific cues. These features of task-specific performance schemas reflect raters’ efforts to understand the requirements of task-specific performance and the use of ‘task-specific performance theory’ to interpret and evaluate what is happening during the patient encounter.

*“This patient is very demanding. And then you know that he (the student) cannot get away with this. He (the patient) wants to overrule the doctor’s decision, and then he (the student) will also have to overrule … and see what he can do for this patient.” (PP21)*

*“He (the student) says “…” And this is a very important sentence in this case. It makes the patient feel welcome, and this is very important for this patient because he is feeling rather uncomfortable about not having gone to the doctor earlier.” (PP 27)*

### Person schema

Table 2 presents the percentage of raters making inferences about the trainee as well as the type and number of verbal utterances reflecting inferences, per group and per VC, and at T1 and T2. The results show that the majority of raters made inferences about trainees while observing and evaluating their performance, especially with regard to salient behaviours (VC1). Table 2 also shows that raters were most likely to be making inferences at T1, when they were forming their first impressions. All five types of inference processing described by Klimoski and Donahue (2001) appeared to be present in the assessment of trainee performance during single patient encounters. Examples of inferences by different raters for each of the VCs are presented in Table 3.

**Table 2:** Content of person schemas and percentages of raters using person schemas, per videocase, at T1 and T2

	Dermacase (VC1)				Cardiocase (VC2)			
	T1		T2		T1		T2	
	Exp (N = 16)	N-exp (N = 12)	Exp (N = 18)	N-exp (N = 16)	Exp (N = 18)	N-exp (N = 16)	Exp (N = 18)	N-exp (N = 16)
*Percentage of raters making inferences (1.1*–*1.5)*	100	100	61	56	78	94	33	38
Total number of inferences (1.1–1.5)	28	23	23	21	33	37	8	11
Number of inferences regarding								
1.1 Knowledge	1	0	2	0	5	5	3	2
1.2 Personality traits	7	7	7	9	10	14	0	1
1.3 Disposition	1	4	5	3	5	13	1	4
1.4 Intentions	12	9	4	6	5	3	2	2
1.5 Social category	7	3	5	3	8	2	2	2
*Percentage of raters incorporating phase of training in their judgments (frame of reference)*	19	17	28	19	28	31	33	44

Presented are the percentages of raters making inferences about the trainee, and the number of inferences made by all raters, in all and per dimension

**Table 3:** Examples of inferences about the trainee, per videocase

1.1 Inferences regarding knowledge
Cardiocase: Definitely adequate knowledge base; knowledge inadequate; he finds it difficult to apply knowledge in clinical practice
Dermacase: I think that he will perform well on knowledge tests
1.2 Inferences regarding personality traits
Cardiocase: <This trainee is> warm-hearted; sympathetic; timid; friendly; well-behaved; nice person
Dermacase: <This trainee is> rigid; cold-hearted; not empathic; interested
1.3 Inferences regarding disposition
Cardiocase: <This trainee> adopts a clinical approach towards his patients; adopts an open approach; finds it difficult to discuss patients’ feelings and emotions; is too much involved with his own thoughts, as are most young residents; finds it difficult to just sit back and listen to the patient, but he will learn in time
Dermacase: <This trainee> adopts a clinical approach; listens attentively and reacts to others
1.4 Inferences regarding intention
Cardiocase: <This trainee> clearly does not want to make any mistakes with this patient; focuses on adequately handling the biomedical aspects of this patient’s problem
Dermacase: <This trainee> definitely wants to stay in charge; focuses on adequately handling the biomedical aspects of this patient’s problem; this trainee is eager to demonstrate that he can handle this
1.5 Inferences regarding social category
Cardiocase: he clearly just finished his clinical clerkships; he cannot think or act like a general practitioner; he has got the capacity to become a good physician; inexperienced
Dermacase: he is an authoritarian doctor; he is a technical doctor; robot-like; doesn’t seem to take any pleasure in being a doctor; quick, efficient worker
2. Phase of training—frame of reference for judging performance
Well, he is a final year student, so I will have to take this into account, won’t I?

### Rater idiosyncrasy

The results for rater idiosyncrasy are presented in Tables 4 and 5.

**Table 4:** Performance theory and rater idiosyncrasy: percentages of experienced raters (Exp) and non-experienced raters (N-exp), using specific performance dimensions, per videocase, at T1 and T2

Performance dimensions	Dermacase (VC1)				Cardiocase (VC2)			
	T1		T2		T1		T2	
	Exp (N = 16)	N-exp (N = 12)	Exp (N = 18)	N-exp (N = 16)	Exp (N = 18)	N-exp (N = 16)	Exp (N = 18)	N-exp (N = 16)
Think/act as GP	50	42	56	44	5	6	11	13
Doctor-patient relationship^a^	100	92	100	94	94	94	89	82
Establishing/developing rapport	63	75	44	38	72	56	17	13
Demonstrating confidence	13	8	0	6	22	6	22	25
Demonstrating empathic behaviour	31	50	50	44	56	56	0	19
Open approach	44	75	33	44	38	69	0	24
Shared mind 1	50	42	56	69	56	44	44	50
Shared mind 2	0	0	39	63	5	6	39	38
Shared mind 3	0	0	44	63	11	0	61	50
Handling (bio)medical aspects^b^	31	33	67	75	61	19	94	81
History taking	19	17	28	56	50	13	22	19
Physical examination	0	0	28	44	0	0	33	32
Diagnosis/DD	0	0	22	25	11	0	33	38
Patient management	0	0	61	31	0	0	72	56
Structuring and time management	13	0	17	50	44	19	22	44

Presented are percentages of raters using a performance dimension for each group of raters and per VC, at T1 and T2 respectively

^a^Doctor-patient relationship: includes main performance dimension “doctor-patient relationship” plus all related sub dimensions

^b^Handling (bio)medical aspects: includes main performance dimension “handling (bio)medical aspects” plus all related sub dimensions

**Table 5:** Performance theory and rater idiosyncrasy: identification of performance-related behaviours by experienced raters (Exp) and non-experienced raters (N-exp), for each performance dimension, per videocase, at T1 and T2

Performance dimensions	Dermacase (VC1)				Cardiocase (VC2)			
	T1		T2		T1		T2	
	Exp (N = 16)	N-exp (N = 12)	Exp (N = 18)	N-exp (N = 16)	Exp (N = 18)	N-exp (N = 16)	Exp (N = 18)	N-exp (N = 16)
	Eff/Ineff	Eff/Ineff	Eff/Ineff	Eff/Ineff	Eff/Ineff	Eff/Ineff	Eff/Ineff	Eff/Ineff
Think/act as GP	0/8	0/5	0/10	0/7	0/1	0/1	0/2	1/1
Doctor-patient relationship total^a^	4/60	1/47	5/77	7/81	71/44	39/25	12/51	18/62
Establishing/developing rapport	4/14	1/11	0/9	0/9	28/5	13/3	3/1	1/1
Demonstrating confidence	0/2	0/1	0/0	0/1	1/4	0/2	0/4	1/7
Demonstrating empathic behaviour	0/7	0/8	0/10	0/9	15/4	11/2	0/0	2/2
Open approach	0/11	0/13	1/8	0/9	17/1	11/4	0/0	3/2
Shared mind 1	0/12	0/8	0/14	0/18	2/17	1/10	1/11	2/16
Shared mind 2	0/0	0/0	2/13	6/13	0/1	0/1	3/8	0/15
Shared mind 3	0/0	0/0	2/13	1/15	0/3	0/0	5/22	8/13
Handling (bio)medical aspects total^b^	8/0	5/1	12/28	25/16	21/2	3/0	17/37	12/32
History taking	5/0	2/1	5/2	12/1	16/1	2/0	3/5	7/2
Physical examination	0/0	0/0	0/9	3/9	0/0	0/0	5/4	5/10
Diagnosis/DD	0/0	0/0	3/1	3/2	1/1	0/0	2/5	1/7
Patient management	0/0	0/0	1/19	4/4	0/0	0/0	5/23	8/13
Structuring and time management	2/1	0/0	1/2	2/9	7/5	0/3	3/4	6/7

Presented are absolute numbers of verbal utterances concerning (effective/ineffective) behaviours within performance dimensions, for each group of raters and per VC, at T1 and T2 respectively

^a^Doctor-patient relationship total = sum of all verbal utterances within the main performance dimension “doctor-patient relationship” and all related sub dimensions

^b^Handling (bio)medical aspects total = sum of all verbal utterances within the main performance dimension “Handling (bio)medical aspects” and all related sub dimensions

Table 4 shows the percentage of raters using a specific performance dimension when rating trainee performance at T1 and T2, for each group of raters (experienced and non-experienced) and for each of the videocases. Very high or very low percentages (close to 100 or 0 %) indicate high levels of between-rater agreement (low levels of rater idiosyncrasy). The closer a percentage moves to 50 %, though, the more raters differ with respect to use of the specific performance dimension, indicating high levels of rater idiosyncrasy. Table 4 shows that (nearly) all raters used the main performance dimension’doctor-patient relationship’ or at least one of its sub-dimensions in both VCs.

For all other (sub-)dimensions the percentages of raters using the dimension varied (often far from 0 or 100 %), indicating considerable between-rater differences in the use of performance theory (i.e. rater idiosyncrasy) during assessment of trainee performance. No consistent relationship was found for between-rater differences and rater expertise.

Table 5 presents the number of verbal utterances concerning effective and ineffective trainee behaviours per performance (sub-) dimension, for each group of raters, for each VC at T1 and T2. Table 5 shows that, in general, raters’ judgments included fewer statements on ‘handling biomedical aspects of the consultation’ compared to ‘doctor-patient relationship’. Especially at T1 (first phase of the consultation), judgments on trainee performance were mainly based on evaluation of performance within the dimension ‘doctor-patient relationship’. The more balanced pattern of effective and ineffective performance-related behaviours in VC2 (cardiocase) reflects the more complex and differentiated behaviours of the trainee in this video. In general, however, raters seemed to pay more attention to ineffective behaviours (negative information) than to effective behaviours at T2, when they gave an overall judgment of trainee performance after viewing the entire VC.

### Rater expertise and the use of task-specific schemas

Results with respect to the use of task-specific schemas are presented in Table 6. Experienced raters paid significantly more attention to task-specific factors in assessing trainee performance. For the complex cardiac case (VC2), significant between-group differences were found with respect to the number of task-specific performance elements (A1 + A2 + A3) per rater at T1 and T2 (*U* = 77.5, *p* = .02, *ES* = .41 and *U* = 86, *p* = .04, *ES* = .35). For the dermatology case (VC1), similar and near-significant differences were found at T1 (*U* = 57, *p* = .07). At T2, significant between-group differences were found for task-specific elements (A1 + A2) (*U* = 73, *p* = .01, *ES* = .44). Although statements about task-specific factors in general accounted for a relatively small percentage of all verbal utterances, Table 6 clearly shows that statements related to task-specific performance schemas represent a substantial part of the verbal protocols of the more experienced raters, and are less frequently used by the group of less experienced raters.

**Table 6:** Task-specific schema: use of task-specific performance schemas by experienced (Exp) and non-experienced (N-exp) raters, per videocase, at T1 and T2

	Derma case (VC1)				Cardiocase (VC2)			
	T1		T2		T1		T2	
	Exp (N = 16)	N-exp (N = 12)	Exp (N = 18)	N-exp (N = 16)	Exp (N = 18)	N-exp (N = 16)	Exp (N = 18)	N-exp (N = 16)
A1 case -specific cues (clinical presentation; patient behaviour; setting consultation)	69 (9.8/13.1)	42 (.0/8.9)	72 (4.9/9.4)	31 (.0/5.5)	67 (7.2/9.3)	38 (.0/9.1)	72 (8.5/14.6)	56 (1.1/8.2)
A2 specific trainee behaviours	44 (.0/7.1)	17 (.0/.0)	44 (.0/7.1)	13 (.0/.0)	44 (.0/7.3)	13 (.0/.0)	56 (6.7/11.3)	38 (.0/5.0)
A3 effects of trainee behaviours	19 (.0/.0)	8 (.0/.0)	22 (.0/.8)	31 (.0/6.1)	44 (.0/8.4)	0 (–)	50 (1.6/7.9)	31 (.0/5.0)
A1-3 task-specific features total	81 (11.8/14.2)	50 (3.6/9.1)	72 (10.6/17.9)	56 (6.3/11.8)	78 (12.9/12.8)	38 (.0/13.8)	78 (20.7/23.6)	69 (6.7/17.2)

Presented are the *percentages* of raters using task- or event-specific elements of performance, and percentages of statements per protocol in parentheses (median/interquartile range), for each group of raters and per VC, at T1 and T2 respectively

## Discussion

Using theoretical frameworks from social perception research, we sought to better understand underpinnings of work-based assessment outcomes by exploring the content of schemas and their use by raters during assessment of trainee performance in single patient encounters. The findings indicate that raters used different schemas interactively: performance theories, task-specific performance schemas and person schemas were used to arrive at judgments. Our results indicate, however, substantial between-rater differences in the use of performance theories (i.e. rater idiosyncrasy) and ‘expert-novice’ differences in the use of task-specific performance schemas.

We used think-aloud procedures during actual rating tasks, which enabled us to establish dimensions of performance used by GP-raters during performance assessment. The performance dimensions in Fig. 2 emerged from the analysis of think-aloud procedures of 34 GP-supervisors rating the performance of two different trainees each conducting a different patient encounter. Performance dimensions and sub dimensions *together* could be considered to reflect a normative performance theory, or ‘performance schema’, of physician performance in general practice, built upon what ‘raters actually pay attention to and comment upon in practice’.

The results from our study seem to be inconsistent with previous research on WBA indicating that raters have a one- or two-dimensional conception of professional competence (‘cognitive/clinical’ and ‘humanistic/(psycho)social’) and are therefore unable to discriminate between different competencies or dimensions (Cook et al. 2010; Pulito et al. 2007; Archer et al. 2010). This so-called halo effect is generally attributed to rater error, resulting from global impression formation, categorization or ‘stereotyping’. The results from our study clearly show that raters distinguished a fairly large number of different performance dimensions and used dimensions interactively when assessing performance. For example, when assessing performance during history taking, physical examination or patient management, raters assessed not only students’ ability to adequately handle (bio)medical or ‘medico-technical’ aspects of the problem, but also their communication and interpersonal as well as time management skills. In other words, the performance theory (or competency framework) used by the raters does not map neatly onto the frameworks of most standardized rating scales, which present performance dimensions as strictly separate, distinct entities (e.g. the typical mini-CEX format). True correlations between different performance dimensions may be high, and observed halo effects may—at least partially—be considered as ‘true halo’ rather than as the result of rater incompetence or automatic top-down categorization of trainee performance.

Our findings also show that GP-supervisors differed in the dimensions they used in performance assessment, indicating varying levels of rater idiosyncrasy. Furthermore, raters used different dimensions, depending on what they actually saw during the patient encounter: apparently not all dimensions are equally relevant or important in all cases. In general, standardized rating scales are designed to represent a given set of performance dimensions (or competencies) in a predefined order, suggesting equal importance of each performance domain. Requiring raters to fill in a rating score for all performance dimensions may therefore hinder accurate depiction of trainee performance. Our findings are in line with findings from Ginsburg et al. (2010), who found that dimensions took on variable degrees of importance, depending on the resident that was being evaluated.

The present study confirms findings of expertise research indicating that, when handling complex tasks, ‘experts’ pay more attention to contextual or situation-specific factors before deciding on a plan of action or solution (e.g. Ross et al. 2006). When assessing student performance in patient encounters, experienced GP-raters paid (significantly) more attention to task-specific cues. Furthermore, experienced raters seemed to be more likely than inexperienced raters to explicitly link task- or case-specific cues to specific trainee behaviours and to effects of trainee behaviour on both the patient and the outcome of the patient consultation. Similar results were found in a study on teacher supervision, in which experienced supervisors, more so than inexperienced supervisors, automatically looked for coherence and meaning in teacher behaviours. Experienced supervisors searched for student involvement and effects of teacher behaviours on student learning, rather than focused exclusively on discrete aspects of teacher behaviours (Kerrins and Cushing 2000). Our findings thus suggest that experienced raters have more differentiated performance schemas, which are activated by task-specific cues. In this respect, our findings are consistent with previous research in industrial and organizational psychology showing that experienced raters are more sensitive to relevant ratee behaviours and have more, and more sophisticated, performance schemas (Cardy et al. 1987).

Findings from our study clearly indicate that raters started to develop person schemas from the moment they began to observe trainee performance. Raters not only made inferences about knowledge and disposition based on what they knew about the trainee (phase of training, for instance), but at least some raters also seemed to categorize trainees according to personality judgments and behavioural interpretations. Although our findings show consensus among raters with respect to some inferences about individual trainees, there was also considerable disagreement. These findings are in line with person perception research, which consistently shows that perceivers’ <idiosyncratic> interpretive processes may produce sharp differences in person perception (Mohr and Kenny 2006). In general, people make social inferences spontaneously (Uleman et al. 2008; Macrae and Bodenhausen 2001), and raters’ person schemas—once developed—may guide (selective) attention in subsequent assessments and colour the interpretation of future information. Differences in the way raters form person schemas in WBA contexts may therefore be one of the major factors underlying differences in rating outcomes.

### Limitations of our study

This study has several limitations. Since all participants were volunteers, they may have been more motivated to carefully assess trainee performance. Combined with the experimental setting of our study, this may limit generalization of our findings to raters in ‘real life’ general practice. Real life settings are usually characterized by time constraints, conflicting tasks, and varying rater commitment, which may all impact on rater information processing. Another limitation of our study may be that the raters were all selected within one geographical region. As a consequence, the normative performance theory that evolved from our data may reflect the structure of assessment tools that are used in local training and GP-supervision practices, thereby limiting generalization of the results to other regions or disciplines. Nevertheless, one of the main findings of our study remains that the raters showed considerable levels of idiosyncrasy, despite their training and considerable experience.

A further limitation is the way we selected the experts. We used only years of experience as a measure of expertise; other variables such as actual supervisor performance, commitment to teaching and assessment, or reflectivity were not measured or controlled for. However, time and experience are clearly important variables in acquiring expertise. In this respect, our relative approach to expertise is very similar to approaches in expertise research in the domain of clinical reasoning in medicine (Norman et al. 2006).

In the setting of our experiment, participants were asked to think aloud while judging trainee performance. The task of verbalizing thoughts while filling out a rating scale and providing a performance score may have introduced an aspect of accountability into the rating task, with both experienced and non-experienced raters feeling compelled to retrospectively explain and justify their actions. However, since providing motivations for any performance rating while giving feedback and discussing ratings with trainees is a built-in characteristic of performance evaluation in general practice, our experimental setting comes close to real life task performance, and verbal protocols in our study most likely reflect ‘natural’ cognitive processing by raters in “context-free” assessment of performance.

### Implications of our study

The results of our study have several implications for WBA practice as well as for future research.

Firstly, our findings may have implications for rater training, providing further support for the implementation of ‘frame-of-reference’ (FOR) training as proposed by Holmboe (2008). As indicated before, results of rater training are often disappointing and one of the major reasons may be that rater training tends to focus on how to use predefined and standardized assessment instruments, ignoring raters’ a priori performance theories. As a consequence, transfer of training may be limited. FOR training on the other hand asks raters to reflect on their personal methods of evaluating performance, and aims to reduce idiosyncratic rating tendencies through discussing and defining performance dimensions, performance-related behaviours and performance levels. FOR training, in other words, establishes a ‘shared mental model’ or ‘shared performance theory’ for observing and evaluating performance. In the performance appraisal domain, FOR training has emerged as the most promising approach to rater training and it has been successfully applied in field settings (Sulsky and Kline 2007; Holmboe et al. 2004).

Secondly, our findings may have implications for the way we select raters in the context of WBA. Based on the findings from our study, the use of task-specific performance schemas by more experienced raters may affect feedback given to learners/trainees. The incorporation of contextual cues by experienced raters can result in qualitatively different, more holistic feedback, focusing on a variety of issues and giving meaning to what is happening in the patient encounter by integrating different aspects of performance. Furthermore, research in industrial and organizational psychology indicates that more experienced raters who use more differentiated performance schemas provide more accurate ratings (e.g. Cardy et al. 1987; Ostroff and Ilgen 1992). Although we did not aim to investigate the relationship between the use of schemas and rating accuracy, our findings point to a need for further research into effects of rater expertise on the accuracy of work-based performance assessment.

The results may also have implications for the design of rating scales or rating formats in WBA. As indicated before, correct interpretation of rating scores and usefulness of performance ratings may be compromised when rating scales do not adequately mirror raters’ performance theories. Eliciting “performance theory-in-use”, as in our experimental setting or as part of FOR-training procedures, may contribute to the development of assessment frameworks and instruments, reflecting what experienced practitioners consider to be of importance in the judgment of trainees. It is to be expected that the use of rating instruments that are in line with raters’ natural cognitive processing and competency frameworks will generate more valid and authentic performance ratings, thereby improving the usefulness of WBA results.

More importantly, however, we feel that our findings illustrate the importance of narrative, descriptive feedback in WBA. From our findings, it is clear that a simple score on a rating scale merely represents the tip of the iceberg of the complex and idiosyncratic information processing by raters. Meaningful interpretation of performance scores therefore requires additional narrative comments providing insight into raters’ personal motivations and argumentations. Narrative feedback and comments will thus support credible and defensible decision making about competence achievement. Moreover, narrative feedback—provided it is provided in a constructive way—is the only way to help trainees to accurately identify strengths and weaknesses in their performance and to effectively guide their competence development.

Finally, the development and use of person schemas may pose a threat to the validity of WBA results (e.g. risk of stereotyping). It is important to realize, however, that schema-based processing in performance assessments is likely to be inevitable: use of schemas helps raters to efficiently process and organize information about ratees. Therefore, efforts to improve WBA should be directed at designing assessment environments in which any unintended effects of schema-based processing are countered. First of all, it seems important for raters to be aware of and recognize the processes by which they form impressions of trainee performance. This requires training, feedback and reflection on performance rating as well as interactions with others involved in the assessment process. More importantly, however, there is recent evidence that social-cognitive processes that underlie judgments (for example the application of stereotypes) are extremely malleable and adaptive to the perceiver’s social goals, motives, emotional state and relationships with others (Smith and Semin 2007). In other words: activation and application of mental representations or knowledge structures, such as person schemas, formerly thought to be subconscious and automatic, are influenced by the social context in which judgments are made. Based on research in work settings in other domains, effective interventions include allocation of adequate resources (time and money) and providing raters with adequate opportunities to observe and evaluate trainees; ensuring prolonged engagement; holding raters accountable for their decisions; and underscoring mutual interdependence between supervisor and trainee (Operario and Fiske 2001). Trustworthiness and rigour of decision making can furthermore be achieved through careful design of decision making strategies, such as ‘critical dialogue’ between different raters/assessors (Van der Vleuten et al. 2010; Moss 1994).

### Conclusive remarks

We feel that the findings of our study contribute to a better understanding of the processes underlying work-based assessments in the clinical domain. When assessing performance, raters make use of personal constructs and theories about performance that develop through prolonged task experience. Idiosyncratic use of performance theories as well as person models that raters arrive at during observation and assessment determine rating outcomes. We conclude that our findings support approaches to WBA from a social-psychological perspective, considering raters to be active information processors embedded in the social context in which assessment takes place.

Further research should examine whether our findings can be reproduced in other settings and other medical specialties. Important areas for research are the use and development of person schemas and their impact on supervisor behaviour towards trainees, feedback processes and subsequent performance evaluations. Further research should address the development of performance schemas over time and consequences for assessment instruments, rater training and rater selection. Clearly, what we need first and foremost are field studies investigating how contextual factors influence the development and use of schemas by raters, and how they affect rating outcomes.
